# Coapplication of Lidocaine and Membrane-Impermeable Lidocaine Derivative QX-222 Produces Divergent Effects on Evoked and Spontaneous Nociceptive Behaviors in Mice

**DOI:** 10.1155/2014/628729

**Published:** 2014-11-24

**Authors:** Si-Ping Hu, Jing-Jing Zhao, Wei-Xing Wang, Yang Liu, He-Fen Wu, Chao Chen, Liang Yu, Jing-Bing Gui

**Affiliations:** ^1^Department of Anesthesiology, Huzhou Central Hospital, Huzhou 313000, China; ^2^Department of Orthopedics, Huzhou Central Hospital, Hongqi Road No. 198, Huzhou 313000, China

## Abstract

The present study was aimed at investigating the analgesic properties of a combination of lidocaine and QX-222 and its effects on evoked pain behavior (complete Freund's adjuvant-induced allodynia and hyperalgesia in inflammatory condition) and spontaneous pain behavior (formalin-induced acute pain) in mice. Drugs were injected adjacent to sciatic nerve or into plantar. Motor function, thermal withdrawal latency, mechanical withdrawal threshold, and licking/biting were evaluated by behavioral tests. A combination of lidocaine and QX-222 adjacent sciatic nerve injection produced the long-lasting sensory-specific nerve block, and intraplantar injection inhibited spontaneous pain in the formalin-treated mice but did not detectably attenuated hyperalgesia and allodynia in the complete Freund's adjuvant- (CFA-) treated mice. Our results suggest that a combination of lidocaine and QX-222 achieves a long-lasting differential block (sensory selective) and produces divergent effects on evoked and spontaneous pain behaviors in mice.

## 1. Introduction

Differential block (of note, especially in the clinical anesthesia literature, the terms differential block and sensory selective are commonly used and are roughly interchangeable with pain selective and nociceptor selective.) has long been a key goal in the clinical anesthesia, greatly depending on the development of novel drugs and new strategies. Under physiological conditions, most local anesthetics (LAs) in clinical use are present in a mixture of protonated and uncharged hydrophobic forms, but only the uncharged base form of LAs can penetrate the membrane of all neurons, blocking the generation and propagation of action potentials via action on an intercellular site at voltage-gated Na^+^ channels [1]. So in addition to blocking voltage-gated Na^+^ channels in sensory nerve, LAs currently applied in peripheral nerve block also block voltage-gated Na^+^ channels in motor and sympathetic nerve [2–6]. Therefore, LAs produce complete pain relief and also cause low blood pressure, loss of innocuous sensation, and motor paralysis resulting of concomitant autonomic, low-threshold sensory and motor fibers block.

Recently, a report demonstrated that the charged, membrane-impermeable lidocaine derivative, QX-314, could be targeted on nociceptors to produce a pain-specific local anesthesia via activated TRPV1 channels by capsaicin [4]. TRPV1 channels are expressed peripherally only in primary afferent nociceptors. In addition to capsaicin, heat (>43°C), protons (pH < 5.9), and inflammatory mediators [7–10], TRPV1 channels can also be activated by lidocaine [11]; furthermore coapplication of lidocaine and QX-314 produced a long-lasting nociceptive block in rodents. 5 QX-222, a similar compound to QX-314, is one of quaternary lidocaine derivatives with obligate positive charges [12, 13]. Recent findings have indicated that coapplication of chemical membrane permeation enhancers Tween 20 or octyltrimethylammonium bromide and QX-314 or QX-222 also produced the prolonged sensory-selective nerve blockade [14].

According to these findings, we hypothesized that QX-222 also could be selectively targeted on nociceptors and produce sensory-selective blockade via lidocaine activated-TRPV1 channels. Here we showed that this hypothesis was correct, insofar as long-lasting sensory-specific nerve block was achieved by the combination of QX-222 and lidocaine. Surprisingly, and much more crucially, we found coapplication of lidocaine and QX-222 produced divergent effects on evoked and spontaneous nociceptive behaviors in mice.

## 2. Materials and Methods

### 2.1. Animals

Adult male Kunming mice (18–22 g) were provided by Zhejiang University Laboratory Animal Center (Zhejiang, China). Mice were kept in animal housing facilities with controlled relative humidity (20–30%), at the room temperature of 23 ± 2°C, in a 12 h (light on 06:00 to 18:00) light-dark cycle, and fed food and water* ad libitum*. Before experiments, the animals were allowed to habituate to the housing facilities for 3 days and efforts were made to minimize the number of animals and limit distress to the animals. All experimental protocols were according to the Measures for the Administration of Experimental Animals of Zhejiang Province, Zhejiang, China, and the Declaration of National Institutes of Health Guide for Care and Use of Laboratory Animals.

### 2.2. Drugs

Lidocaine hydrochloride and QX-222 (N-(2,6-Dimethylphenylcarbamoylmethyl) trimethylammonium chloride) were purchased from Scent LLC (New Jersey, USA). Normal saline (NaCl) solution was 0.9% wt/vol. Lidocaine and QX-222 were 0.5% wt/vol dissolved in saline when administrated and freshly prepared before the start of the experiments.

### 2.3. Experimental Protocols

The protocol was divided into 2 parts. In part I, the neurobehavioral effects of sciatic nerve blockade of lidocaine (0.5%, 20 *μ*L), QX-222 (0.5%, 20 *μ*L), and lidocaine + QX-222 (0.5%, 20 *μ*L) were measured (*n* = 8 mice for each drug). In part II, spontaneous pain behaviors were evaluated after intraplantar injection of lidocaine + QX-222 (0.5%, 10 *μ*L) in the formalin induced acute pain model (*n* = 8 mice), and evoked pain behaviors were evaluated after intraplantar injection of lidocaine + QX-222 (0.5%, 10 *μ*L) in the CFA induced chronic pain model (*n* = 8 mice). The saline group was used as a control (*n* = 8 mice).

### 2.4. Part I: Sciatic Nerve Injection and Neurobehavioral Examinations

A trained examiner blinded to the experimental groups was responsible for handling the animals and behavioral examinations. The sciatic nerve blockade method is as described by Leszczynska and Kau and Lim et al. with minor modifications [15, 16]. In brief, mice were slightly restrained and 20 *μ*L drugs were injected into the area of the popliteal fossa of left hind limb using a 50 *μ*L Hamilton syringe with a 28-gauge needle. All mice were placed in the middle of a 20 × 25 cm inverted mesh and acclimatized to climb to the outside and over the edge of the mesh, and mice could climb on mesh with all four limbs before experiments. After injection, mice were placed onto the mesh, and primary endpoint was the time to loss of ability to hang on to the inverted mesh with the injected hind limb, which was tested for at 5, 10, 15, and 20 min after injection.

Thermal hyperalgesia was measured by the IITC Plantar Analgesia Meter (IITC Life Science Inc.) for paw withdrawal latency (PWL) according to the method described by Hargreaves et al. [17]. In brief, mice were placed in transparent acrylic enclosures (7 × 9 × 11 cm) with a glass plate and allowed to acclimatize to their environment for 1 h before testing in a temperature-controlled and noise-free room (23 ± 2°C). The high-intensity, movable radiant heat source was placed underneath glass and focused onto the plantar surface of each hind paw. When the animal was at rest, not walking, with its hind paw contacting the glass plate, care was taken to initiate the test. The nociceptive endpoint in the radiant heat test was characteristic lifting or licking of the hind paw. The time from onset of radiant heat to endpoint was considered as PWL. The radiant heat intensity was adjusted at the beginning of the experiment to obtain basal PWL and kept constant thereafter. An automatic 25 s cutoff was used to prevent tissue damage. Each animal was tested 3 times on each hind paw at intervals of 5 min.

Von Frey test was performed for mechanical allodynia. Mice were placed in a plastic cage (20 × 25 × 15 cm) with a plexiglass floor, containing 1.5 mm diameter holes in a 5 mm grid of perpendicular rows throughout the entire area of the platform. After being allowed to acclimate for 1 h, mechanical withdrawal threshold (MWT) was measured as the hind paw withdrawal response to Von Frey hair stimulation according to the up-down method of Dixon. An ascending series of Von Frey hairs with logarithmically incremental stiffness (starting with 0.31 g and ending with 4.0 g filament as cutoff value) (Stoelting, Wood Dale, Illinois, USA) were applied perpendicular to the mid-plantar surface (avoiding the less sensitive) of each hind paw. Each Von Frey hair was held for 1-2 s. Each hair was applied five times at 5 s intervals. After the threshold was determined for one hind paw, the same testing procedure was repeated on the other hind paw at a 5 min interval. A positive response was defined as a brisk withdrawal of hind paw or paw flinching upon stimulus. Whenever a positive response to a stimulus occurred, the next lower Von Frey hair was applied, and whenever a negative response occurred, the next higher hair was applied. The testing consisted of five more stimuli after the first change in response occurred, and the pattern of responses was converted to a 50% Von Frey threshold using the method of Dixon, described by Chaplan et al. [18].

### 2.5. Part II: Intraplantar Injection and Neurobehavioral Examinations

The procedure used was essentially the same as that reported by Hunskaar and Hole [19] and Ruan et al. [20]. Approximately 30 min before testing, mice were individually placed in perspex observation chambers (10 × 20 × 15 cm) for adaptation. Then, the animals were taken out of the chamber, and 10 *μ*L of 1% formalin in 0.9% saline was injected subcutaneously into the dorsal surface of the right hind paw with a 25 *μ*L Hamilton syringe with a 28-gauge needle. Immediately after formalin injection, each mouse was returned to the observation chamber and the behaviors were recorded by a computer-based video recording system for 60 min. The time spent licking the injected paw was calculated in 5 min epochs [21]. Drugs were injected 15 min before formalin injection.

The complete Freund's adjuvant (Sigma, St. Louis, MO) inflammatory model was produced by injecting 10 *μ*L of CFA subcutaneously into the plantar of the left paw. PWL and MWT were assessed 1 day after injection and at 0.5, 1.0, 1.5, and 2.0 h after drugs injection.

### 2.6. Statistical Analysis

Statistical analysis of more than two groups was performed with one-way analysis of variance (ANOVA) followed by Tukey's post hoc test. The significance of any differences in PWL and MWT in behavioral test was assessed using two-way ANOVA. “Time” was treated as a “within groups” factor and “treatment” was treated as a “between groups” factor. Survival curves of categorical time to event data were compared with the log-rank test; survival fractions were calculated using the product limit (Kaplan-Meier) method. Differences were considered significant at *P* < 0.05. Statistical tests were two-tailed, and data were presented as mean ± S.D.,* n* = sample size. Statistical analyses of data were analyzed using Prism version 5 (GraphPad, San Diego, CA). All the tests were performed blind.

## 3. Results

**Figure 1:**
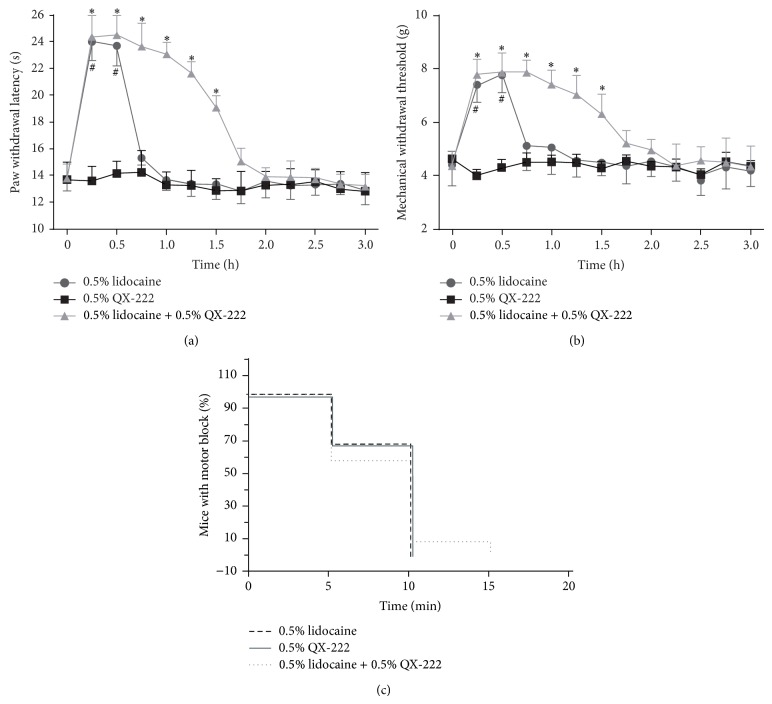
Sciatic nerve block with a combination of 0.5% lidocaine and 0.5% QX-222 produces sensory-specific analgesic effect in naïve mice. Sciatic nerve injection of 0.5% lidocaine or a combination of 0.5% lidocaine and 0.5% QX-222 inhibited thermal hyperalgesia and mechanical allodynia ((a) and (b)), and both exhibited signs of brief motor block (c) in naïve mice. ^*^ *P* < 0.0001, ^#^ *P* < 0.0001 versus 0.5% QX-222 (*n* = 8).

Coapplication of lidocaine and QX-222 produces sensory-selective blockade in the mouse sciatic nerve blockade model (Figure 1). Mice in 3 groups exhibited a similar baseline of the PWL to the Hargreaves test before sciatic nerve blockade. Sciatic injection of QX-222 alone did not affect the PWL to a noxious thermal stimulus. Injection of lidocaine into the area of the popliteal fossa led to a substantial but brief elevation in the PWL (*P* < 0.001), and this effect completely reversed 45 min after lidocaine injection. A combination of lidocaine and QX-222 injected together in immediate proximity to the sciatic nerve produced an increase in the PWL, which is significantly longer than that of lidocaine (*P* < 0.001), the effects of which only reversed fully at 120 min. The data revealed significant effects of sciatic injection of a combination of lidocaine and QX-222 treatment (*P* < 0.0001), time (*P* < 0.0001), and interaction between a combination of lidocaine and QX-222 treatment and time (*P* < 0.0001) (Figure 1(a)). Similar to the effects on PWL, all groups exhibited a similar baseline of the MWT to the Von Frey test before sciatic nerve blockade. Sciatic injection of QX-222 alone did not affect the MWT to monofilament Von Frey hairs stimulus. Injection of lidocaine into the area of the popliteal fossa led to a substantial but brief elevation in the MWT (*P* < 0.001), and this effect completely reversed at 45 min following the injection. A combination of lidocaine and QX-222 injected together in immediate proximity to the sciatic nerve produced an increase in the MWT, which is significantly longer than that of lidocaine (*P* < 0.001), the effects of which only reversed fully at 120 min. Two-way ANOVA revealed significant effects of sciatic injection of a combination of 0.5% lidocaine and 0.5% QX-222 treatment (*P* < 0.0001), time (*P* < 0.0001), and interaction between treatment and time (*P* < 0.0001) (Figure 1(b)). All of the mice injected with Lidocaine, QX-222, or a combination of lidocaine and QX-222 exhibited signs of motor block. Lidocaine or a combination of lidocaine and QX-222 injected together into the popliteal space prevented briefly all mice from using the injected hind limb to hang on to an inverted mesh, which had similar effects on motor nerve. The time to recovery of these mice's ability to hang on to the inverted mesh was 5–10 min (lidocaine and QX-222), and 5–15 min (a combination of lidocaine and QX-222), respectively (Figure 1(c)).

**Figure 2:**
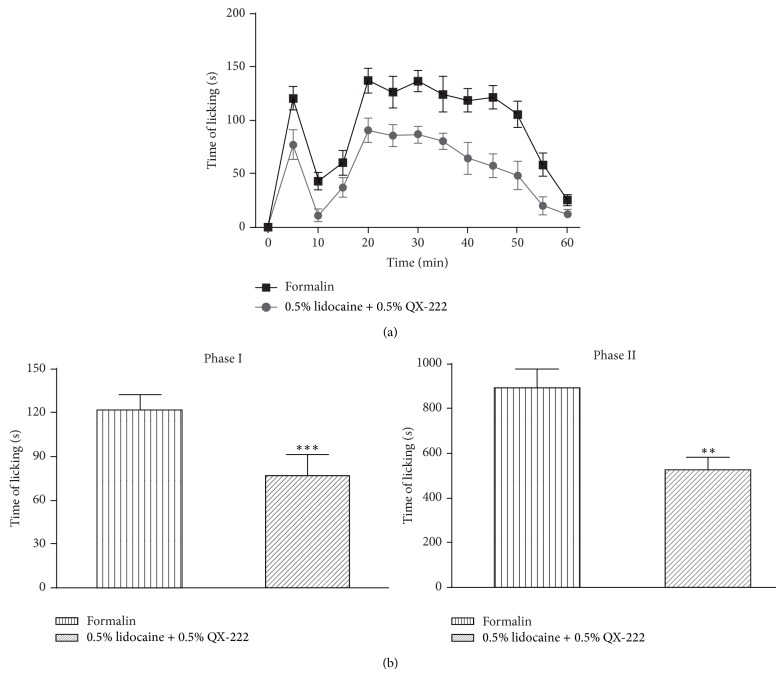
The effects of a combination of 0.5% lidocaine and 0.5% QX-222 on spontaneous pain behaviors. 15 min before treatment, injection of a combination of 0.5% lidocaine and 0.5% QX-222 inhibited spontaneous pain (a). The cumulative licking/biting times of mice in the 0.5% lidocaine + 0.5% QX-222 group were significantly decreased in early and late phase (b). ^***^ *P* < 0.0001, ^**^ *P* < 0.01 versus formalin (*n* = 8).

Intraplantar injection of a combination of lidocaine and QX-222 suppresses the spontaneous pain behavior in the formalin test (Figure 2). Intraplantar injection of formalin produced a typical biphasic pattern of licking/biting behaviors. Throughout the entire hour of observation, compared with mice in the formalin group, the licking/biting behaviors of mice in the lidocaine + QX-222 group were significantly suppressed (*P* < 0.001) (Figure 2(a)). Cumulative licking/biting time of mice in the lidocaine + QX-222 group distinctly showed a decrease in phase I (0–5 min) (*P* < 0.0001) and phase II (15–60 min) (*P* < 0.01) (Figure 2(b)).

**Figure 3:**
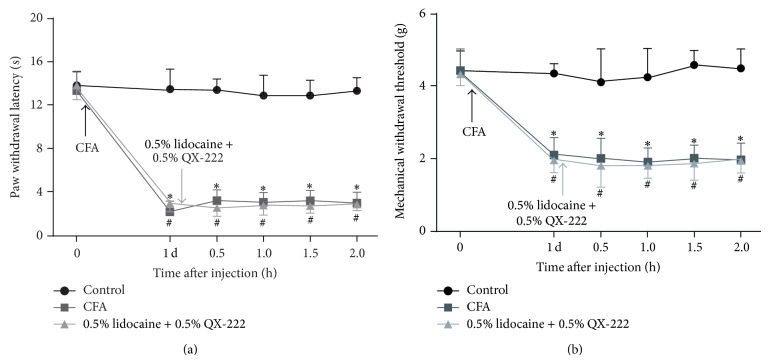
The effects of a combination of 0.5% lidocaine and 0.5% QX-222 on evoked pain behaviors. On the first day after treatment, CFA-treated mice exhibited significant decrease of PWL and MWT; coapplication of lidocaine and QX-222 did not attenuate the thermal hyperalgesia and mechanical allodynia in the CFA-treated mice ((a) and (b)). ^*^ *P* < 0.0001, ^#^ *P* < 0.0001 versus control (*n* = 8).

Coapplication of lidocaine and QX-222 cannot inhibit evoked pain behavior in the CFA model (Figure 3). Mice in each group exhibited a similar baseline of the PWL to the Hargreaves test before treatment. PWL of the CFA-treated mice was accessed on the first day after treatment. CFA-treated mice exhibited significantly shorter PWL than control mice (*P* < 0.001). Coapplication of lidocaine and QX-222 had no detectable effects on the CFA-treated mice (Figure 3(a)). All groups exhibited a similar baseline of the MWT to the Von Frey test before treatment. On the first day after treatment, CFA-treated mice exhibited significant decrease of MWT (*P* < 0.0001). Coapplication of lidocaine and QX-222 did not elevate the MWT in the CFA-treated mice (Figure 3(a)).

## 4. Discussion

In present study, we investigated the analgesic properties of a combination of lidocaine and QX-222 and its effects on spontaneous pain (formalin-induced acute pain) and evoked pain (CFA-induced hyperalgesia and allodynia) in mice. A new finding of this study demonstrated that a long-lasting sensory-specific nerve block was achieved by a combination of QX-222 and lidocaine. Furthermore, coapplication of lidocaine and QX-222 produced divergent effects on evoked and spontaneous nociceptive behaviors in mice.

Almost all LAs in clinical use produce analgesic effects by interrupting nerves excitation and conduction via blockade of voltage-gated sodium channels [22, 23]. Therefore, LAs also impair movement while blocking pain sensation, limiting their clinical application. However, accumulated evidences have shown that QX-314 enters into the nociceptors via activated-TRPV1 or surfactants-induced penetration increase of membrane and blocks the Na^+^ channels from the intracellular side [4, 16]. Furthermore coapplication of lidocaine and QX-314 produced a long-lasting nociceptive blockade in rodents [5]. QX-222, a similar compound to QX-314, is quaternary lidocaine derivatives with obligate positive charges [12, 13]. Recent findings have demonstrated that coapplication of chemical membrane permeation enhancers Tween 20 or octyltrimethylammonium bromide and QX-314 or QX-222 also produced the prolonged sensory-selective nerve blockade [14]. Therefore, we speculated that sufficient QX-222 could enter into nociceptor via the activation of TRPV1 channels by lidocaine and produce a long-lasting sensory-specific nerve block which was followed by the brief nonselective effects of lidocaine injection alone. In our experiment, we find that sciatic injection of 0.5% QX-222 alone did not produce any detectable block, when injected adjacent to sciatic nerve; 0.5% lidocaine produced short-lasting (approximately 10 min) motor in line with its nonselective Na^+^ channel blocker action. Unlike 0.5% lidocaine alone, however, a combination of 0.5% lidocaine with 0.5% QX-222 also significantly prolonged the nociceptive-selective block (approximately 90 min) beyond beginning with a brief nonselective block of the same duration as lidocaine alone.

TRPV1 channel is expressed specifically in primary afferent nociceptors, most of which are unmyelinated, and is physiologically activated and sensitized by heat, protons, and various inflammatory mediators such as bradykinin, adenosine, adenosine triphosphate, and arachidonic metabolites such as lipoxygenase products, leukotriene B4, and prostaglandins, which make up an “inflammatory soup” [24, 25]. To test the analgesic effects of coapplication of lidocaine and QX-222, we employed an acute (spontaneous) and chronic (evoked) inflammatory pain model. Surprisingly, we found that coapplication of lidocaine and QX-222 produced divergent effects on evoked and spontaneous nociceptive behaviors in mice. Namely, unlike effects on the formalin-treated mice, a combination of lidocaine and QX-222 could not inhibit hyperalgesia and allodynia in the CFA-treated mice. We speculated that the probable reason of divergent effects might be desensitization of the TRPV1 channels and metabolization or buffering of QX-222 by “inflammatory soup” in the CFA-treated mice.

In conclusion, a combination of lidocaine with QX-222 injected adjacent sciatic nerve produced the long-lasting sensory-specific nerve block and inhibited spontaneous nociceptive behaviors in the formalin-treated mice; however, the coapplication did not detectably attenuate hyperalgesia and allodynia in the CFA-treated mice.
